# Toward the promotion of *One Health* – Part II: Interdisciplinary research cooperation between digital transformation and exposome

**DOI:** 10.1016/j.jphyss.2026.100078

**Published:** 2026-04-26

**Authors:** Takeshi Yoneshiro, Yoshito Kumagai, Keiko Nohara, Shingo Iwami, Naoki Honda, Nobuhiko Ohno, Motohiro Nishida

**Affiliations:** a Division of Molecular Physiology and Metabolism, Tohoku University Graduate School of Medicine, Sendai, Miyagi 980-8575, Japan; b Division of Metabolic Medicine, Research Center for Advanced Science and Technology (RCAST), The University of Tokyo, Meguro-ku, Tokyo 153-8904, Japan; c Department of Physiology, Graduate School of Pharmaceutical Sciences, Kyushu University, Fukuoka 812-8582, Japan; d Health and Environmental Risk Division, National Institute for Environmental Studies, Tsukuba, Ibaraki 305-8506, Japan; e Graduate School of Life Science, Nagoya University, Nagoya, Aichi 464-8602. Japan; f Nagoya University Graduate School of Medicine, Nagoya, Aichi 466-8550, Japan; g Center for One Medicine Innovative Translational Research (COMIT), Nagoya University, Nagoya 466-8550, Japan; h Graduate School of Integrated Sciences for Life, Hiroshima University, Higashihiroshima, Hiroshima 739-8526, Japan; i National Institute for Physiological Sciences, National Institutes of Natural Sciences, Okazaki, Aichi 444-8787, Japan; j Department of Anatomy, Division of Histology and Cell Biology, Jichi Medical University, Tochigi 329-0498, Japan; k Exploratory Research Center on Life and Living Systems, National Institutes of Natural Sciences, Okazaki, Aichi 444-8787, Japan

**Keywords:** Exposome, Digital transformation, Epigenetics, Drug repurposing, Predictive modeling

## Abstract

Human activities increasingly disrupt global ecosystems, contributing to climate change, biodiversity loss, and emerging health threats. In response, the One Health framework has gained attention as an integrative approach encompassing human, animal, and environmental health. In a symposium at APPW2025, experts in Exposome science and Digital Transformation discussed how interdisciplinary integration can advance predictive and preventive medicine. This review summarizes five key topics: Exposome as a determinant of disease risk, epigenetic mechanisms encoding environmental memory, environmental programming of brown adipose tissue, adaptive prioritization of environmental signals, and simulation-based drug repurposing. Collectively, these studies highlight a paradigm shift from conventional linear exposure–disease models toward a systems-level understanding integrating cumulative exposures, biological memory, and predictive modeling. The convergence of Exposome science and Digital Transformation provides a foundation for advancing One Health into a predictive and actionable scientific framework.

## Highlights

• Integration of Exposome and Digital Transformation advances One Health.
• Epigenetic mechanisms encode environmental memory across generations.
• Simulation-based drug repurposing enables rapid pandemic response.
• Environmental exposures shape long-term metabolic and physiological states.
• Adaptive prioritization reveals dynamic interpretation of environmental signals.

## Introduction

Human health is inextricably linked to the health of animals, ecosystems, and the global environment. Rapid industrialization, urbanization, and globalization since the mid-20th century have profoundly transformed the Earth system, giving rise to the Anthropocene. This era is characterized by climate change, biodiversity loss, widespread chemical pollution, and the emergence of infectious diseases, fundamentally reshaping health risks and necessitating integrated, systems-level approaches.

The "One Health" concept provides a unifying framework recognizing the interconnectedness of human, animal, and environmental health. Within this framework, two rapidly advancing domains - Exposome science and Digital Transformation - are emerging as complementary pillars. Exposome captures the totality of environmental exposures across the life course, while digital technologies, including mathematical modeling and artificial intelligence, enable integration and prediction from complex biological and environmental data.

Importantly, the integration of Exposome science with Digital Transformation represents a conceptual advance beyond traditional environmental health paradigms. Rather than focusing on single exposures, this approach emphasizes cumulative exposures, biological encoding through epigenetic and physiological memory, and predictive modeling for disease prevention and intervention.

**Fig. 1:**
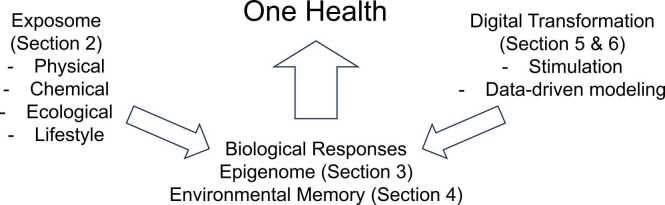
Conceptual framework integrating Exposome science and Digital Transformation within the One Health paradigm. Each component corresponds to Sections 2–6, Sections 2–6, Sections 2–6, Sections 2–6, Sections 2–6 of this review.

This symposium and review highlight five interconnected topics: (1) Exposome as a determinant of disease risk, (2) epigenetic mechanisms encoding environmental memory, (3) brown adipose tissue as a model of environmental memory, (4) adaptive prioritization of environmental signals, and (5) simulation-based drug repurposing. Together, these topics illustrate how interdisciplinary integration can accelerate the realization of One Health (Fig. 1).

## What is exposome? (Yoshito Kumagai)

Concerns are growing over time about increasing health risks. Among the major geological periods, spanning from the Precambrian to the Cenozoic, the Anthropocene is the most recent. It is said to have begun in 1950, marking a period in which humans have dramatically altered the global environment. The Anthropocene is characterized by climate change, including global warming, loss of biodiversity due to mass extinctions, an increase in chemicals, and the combustion of fossil fuels, all of which are believed to be caused by human activity. One hypothesis is that high or chronic exposure to a wide variety of chemicals could result in the crossing of planetary boundaries, or "earth's limits." Planetary boundaries are broadly categorized into three categories, and the most noteworthy among them is the threat posed by humans. For example, there are concerns that the Earth may be reaching its limits due to the chemical load of atmospheric aerosols, including polycyclic aromatic hydrocarbons and aliphatic hydrocarbons, and the contamination of the biosphere by toxic heavy metals and organic matter. Some theories suggest that the Planetary Boundaries may have already begun around 2010. In other words, have we already crossed the threshold?

Based on established causal exposures and diseases, it has been reported that 9 million deaths per year (16% of global deaths) are attributable solely to air, water, and soil pollution. Particulate matter (e.g., PM2.5) and smoking account for just under 30% of exposure risk factors contributing to chronic disease deaths, while surprisingly, unidentified factors account for approximately half of the total (this figure is based on the World Health Organization's estimate that these factors contributed to 50 million deaths worldwide in 2010). This suggests that humans may be ingesting (or being exposed to) combinations of chemicals over long periods of time through their living environment, lifestyle, and dietary habits that are not recognized as dangerous. The economic cost of chemical pollution is significant, with medical care and disability-related productivity losses estimated at US$4.6 trillion per year, equivalent to 6.2% of global economic output. To investigate the global impact of genetic factors on chronic disease risk, we pooled data from a Western European identical twin cohort and calculated population attributable fractions for chronic diseases. For example, the median attributable fraction for cancer was low at 8.3%, compared with 26.1% for neurological diseases and 33.6% for pulmonary diseases. By chronic disease type, the median attributable fraction for leukemia was low at 3.4%, followed by 4.4% for bladder cancer, 9.4% for lung cancer, and 19% for prostate cancer. Among neurological diseases, Parkinson's disease accounted for 9.8%, dementia for 25.2%, and Alzheimer's disease for 32.2%. Among pulmonary diseases, chronic obstructive pulmonary disease accounted for 18.5% and asthma for 48.8%. Overall, these results suggest that environmental factors (nurture) contribute more to chronic disease risk than genetic factors (birth). For these reasons, there is growing recognition that non-genetic factors play a central role in disease development, and the importance of characterizing these factors on a scale comparable to the human genome is recognized.

**Fig. 2:**
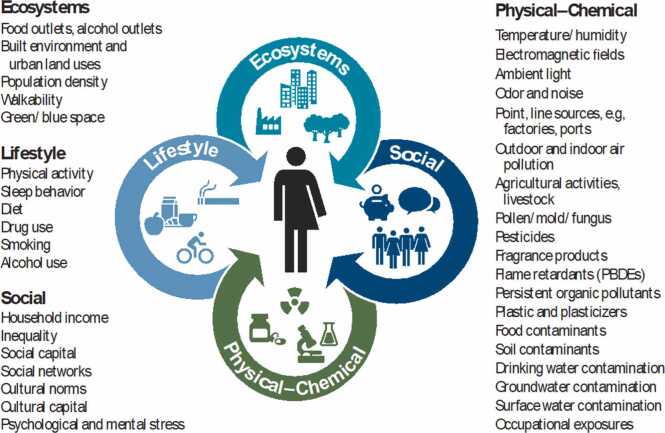
The Exposome concept cited from Vermeulen R et al. Science 2020 [1].

As recognition of the central role of non-genetic factors in disease development grew, Christopher Wild, former director of the International Agency for Research on Cancer (IARC), coined the term "Exposome" in 2005, laying the foundations for the concept. The term "Exposome" is a combination of "exposure" and "ome," and encompasses environmental exposures throughout life, including lifestyle factors, beginning before birth. While the Exposome is an appealing concept, it is broad and difficult to grasp as a research subject. To address this situation and promote Exposome research, Wild classified environmental factors into general external factors, internal factors, and specific external factors in 2012. However, environmental factors are now sometimes classified as physical/chemical factors, ecological factors, lifestyle factors, and social factors (Fig. 2). Besides Wild, Dean Jones of Emory University and his student Gary Miller, now at Columbia University, are also key figures in the advancement of Exposome research [1]. They led Exposome research from exposure science to biology. Miller, in particular, not only published the book "The Exposome" but also contributed to the publication of the academic journal "Exposome". They emphasized a clear distinction between Wild's definition and the Exposome, arguing that it is more appropriate to compare it to the genome, the foundation of modern medicine and public health. The Exposome is a cumulative biological response concept that considers environmental responses, adaptations, and decays in response to chemical intrusions into the body. This includes changes in gene expression via intracellular signaling pathways and epigenetic changes such as protein methylation and acetylation. It also emphasizes endogenous processes such as metabolic activation of ingested chemicals and detoxification by various enzyme proteins. While the Exposome is a fascinating concept, its broad scope and the difficulty of grasping it as a research subject have hindered research progress. However, limited Exposome studies that included blood, food, air pollution, ecosystems, and chemicals as a preliminary step to the Exposome have led to breakthroughs, and research in this field is rapidly progressing. The greatest challenge in Exposome research is not only the sheer number of chemicals of interest, but also the lack of established analytical methods for them, leaving their biological effects unknown. Consequently, determining which chemicals to prioritize for Exposome analysis has become a common issue. In essence, rather than randomly studying every chemical being synthesized and manufactured, we should select and study chemicals directly related to health hazards. Therefore, electrophiles have emerged as promising priority test substances for Exposome research.

**Fig. 3:**
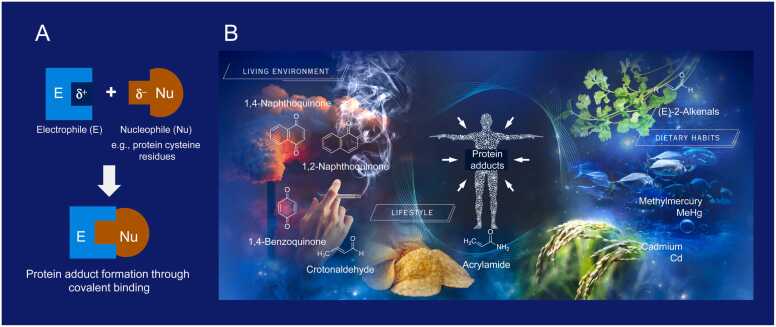
Electrophilic stress: Multiple exposure of humans to a variety of electrophiles in daily life.

Electrophiles contain low-electron-density sites within their molecules, which covalently bond with high-electron-density nucleophilic molecules, such as cysteine residues in proteins, to form stable adducts (Fig. 3A). We are exposed to a variety of electrophiles on a daily basis through our living environment, lifestyle, and diet (Fig. 3B). Representative examples include naphthoquinones present in PM2.5 and the volatile fraction of air, 1,4-benzoquinone and crotonaldehyde present in cigarette smoke, acrylamide present in potato chips and coffee, cadmium present in rice, methylmercury that accumulates in large edible fish such as tuna, and (*E*)-2-alkenals present in high concentrations in coriander leaves such as cilantro. Although these substances differ in structure, they all possess electrophilic properties, and when they enter the body, they form adducts with nucleophilic functional groups on proteins, causing electrophilic stress. To understand the relationship between electrophilic stress and health or disease, our research group exposed purified proteins, cultured cells, and mice individually to the above environmental electrophiles. The results suggest that, at low doses, environmental electrophiles covalently bind to sensor proteins in redox signaling pathways such as PTP1B/EGFR signaling, Keap1/Nrf2 pathway, HSP90/HSF1 signaling, and PTEN/Akt signaling, involved in cell proliferation, oxidative stress response, protein quality control, and cell survival, thereby activating transcription factors and kinases associated with cellular adaptive response to such reactive chemicals. On the other hand, high-dose exposure revealed nonspecific chemical modification of intracellular proteins, resulting in toxicity. Next, in light of the limited Exposome study, we examined combined exposure to environmental electrophiles. The results showed that combined exposure to environmental electrophiles lowered the threshold for dose-dependent activation and disruption of the intracellular redox signaling pathways observed with single exposure, and additive and synergistic effects were observed in terms of toxicity. This suggests that even low exposure to each environmental electrophile can produce unexpected harmful effects.

Taken together, Exposome research highlights the dominant contribution of environmental factors to disease risk. However, a key unresolved question is how such exposures are biologically encoded and retained over time. This leads to the concept of the epigenome as a molecular interface that translates environmental signals into long-lasting biological effects.

## Exposome and epigenome (Keiko Nohara)

Human and animal health is affected not only by genetic factors but also by non-genetic factors, including a wide range of environmental exposures that are increasingly recognized as significant determinants of health. The concept of the Exposome emphasizes the importance of considering exposure to non-genetic factors throughout the life course [2] or, as described below, including those of before conception. It aims to analyze environmental exposures at an omics scale and integrate them with health outcomes and multi-omics data on biological responses.

**Fig. 4:**
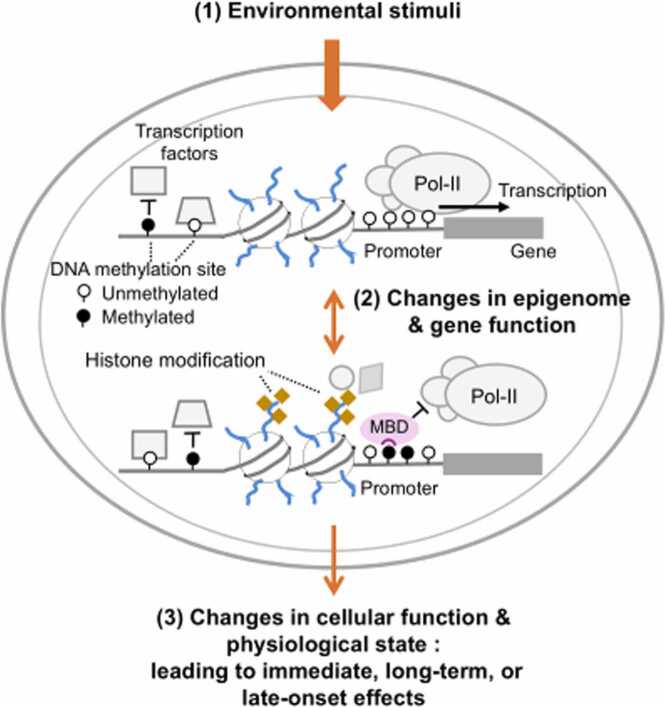
The epigenome is altered by environmental factors. These changes can lead to adverse health effects.

The epigenome, which comprises epigenetic modifications to the genome such as DNA methylation, histone modifications, and non-coding RNA production, plays a crucial role in regulating genomic functions [3]. Unlike the genome, the epigenome can be reversibly altered in response to internal stimuli. This flexibility allows cells with the same genome to differentiate into diverse cell types. Similarly, the epigenome responses to environmental factors. Such changes can be maintained and serve as biological memory, contributing to long-term or late-onset effects that appear after some time or even years later. Excessive environmental stress may disrupt the epigenome, leading to abnormal physiological responses and diseases [4]. Thus, the epigenome plays a role as a pivotal interface between environmental effects and biological reactions in humans and animals. It represents one of the keys to a comprehensive understanding of interactions between living organisms and their environment (Fig. 4).

**Fig. 5:**
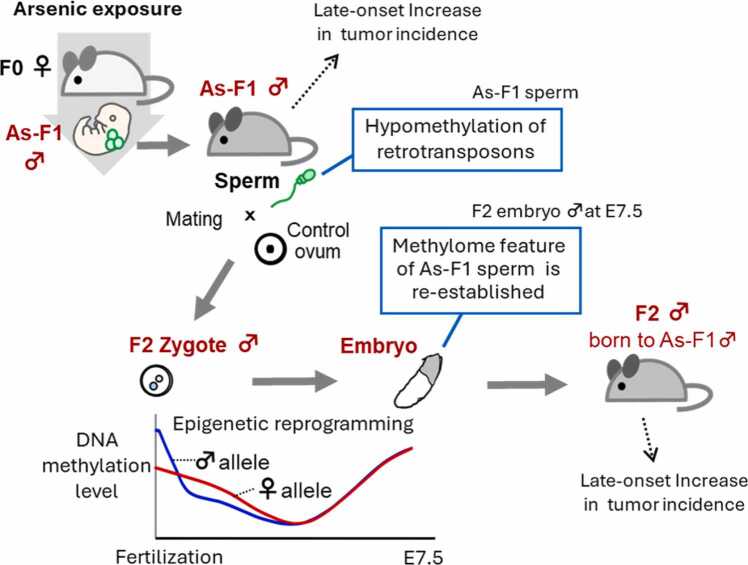
Methylome features acquired in F1 sperm by gestational arsenic exposure are re-established in F2 embryos during epigenetic reprogramming. Please see in the main text for details.

DNA methylation or methylome in gametes, that is, sperm and ova, is essential for proper development following fertilization [5]. After fertilization, the zygotic epigenome undergoes extensive epigenetic reprogramming, during which DNA methylation is largely erased to establish totipotency, and subsequently re-established (Fig. 5). For a long time, it was believed that environmentally induced changes in methylome were eliminated during epigenetic reprogramming, thus environmental exposures did not affect the offspring. However, over the past two decades, research has revealed that exposure to adverse environmental factors can induce epigenetic modifications in parental gametes, leading to health effects on offspring. Furthermore, numerous studies have suggested that environmental exposures of male parents alone can affect their offspring and, in some cases, even subsequent generations through modifications in the sperm epigenome [6]. These findings underscore the importance of the preconception period, during which the epigenome is established, for later-life health outcomes of offspring.

Fig. 5 illustrates the findings from studies on the paternal inheritance of health effects induced by arsenic exposure. Inorganic arsenic is widely distributed in the environment and poses serious global health risks, particularly in highly contaminated regions around the world. A previous study demonstrated that exposure of pregnant C3H mice (F0) to arsenic increased the incidence of hepatic tumors in F1 male offspring [7] (Fig. 5). C3H mouse is a strain whose males are predisposed to spontaneously develop hepatic tumors later in life. Using this model, we found that F2 males born to arsenic-exposed F1 males exhibited a higher incidence of hepatic tumors than F2 males born to the control F1 males [8] (Fig. 5).

The epigenome of fetal germ cells also undergoes reprogramming. We investigated whether arsenic exposure during gestation affects the F1 sperm methylome and whether such alterations are transmitted to F2 embryos, overcoming epigenetic reprogramming at zygotic development. The studies using one-base resolution analysis showed that the sperm methylome of arsenic-exposed F1 males exhibited distinctive features, including enrichment of hypomethylation at the promoters of retrotransposons such as LINEs and LTRs [9]. Hypomethylation of retrotransposons potentially augments harmful retrotransposition. Furthermore, we clarified that similar methylome features of arsenic-exposed F1 sperm, including hypomethylation of retrotransposon promoters, were recapitulated in both paternal and maternal genomes of embryos after epigenetic reprogramming [10] (Fig. 5). These findings support the notion that environmental exposures in male parents can affect offspring health through epigenetic modifications.

Lifelong Exposome analysis remains a major challenge. Considering exposures that occur before conception and even during the fetal stage of the parents adds further complexity. However, the extent to which these emerging concerns contribute to human health is yet to be clarified. To fully realize the Exposome concept, further studies are required to prioritize the factors that should be taken into account.

While epigenetic mechanisms provide a framework for understanding how environmental exposures are stored as biological memory, translating these insights into disease prevention and therapeutic strategies requires integrative and predictive approaches. In this context, Digital Transformation, including simulation-based methodologies, offers powerful tools for bridging molecular insights with clinical applications.

## Brown adipose tissue, environmental memory, and energy homeostasis (Takeshi Yoneshiro, Mami Matsushita, Sayuri Fuse-Hamaoka, Takafumi Hamaoka, Masayuki Saito, Juro Sakai)

### Introduction

Adaptive thermogenesis is a fundamental homeostatic process and crucial for survival in cold environment in mammals. Regulation of nonshivering thermogenesis in brown adipose tissue (BAT) in response to cold exposure differs between acute and chronic activation. Acute cold exposure (*i.e.* a few minutes) evokes immediate heat generation in BAT through the activation of the sympathetic nervous system. In contrast, prolonged cold exposure (*i.e.* several weeks) induces *de novo* brown adipocyte differentiation in BAT and the appearance of beige adipocytes within white adipose tissue, resulting in beneficial metabolic effects such as an increase in energy expenditure (EE) and a decrease in body fat content [11]. The physiological relevance of BAT in adult humans has been suggested by studies showing negative associations of BAT activity with visceral fat accumulation, insulin resistance, and cardiometabolic health [11], [12], indicating that BAT is a potential therapeutic target to mitigate metabolic disorders. Indeed, there have been substantial efforts to establish agents with the capacity to stimulate BAT thermogenesis [11]. In order to harness the potential of BAT for metabolic health, the research filed must move beyond these agents and focus on elucidating how inter-individual difference and age-associated decline in BAT activity are programmed in humans.

### Transgenerational program of thermogenic activity of BAT

**Fig. 6:**
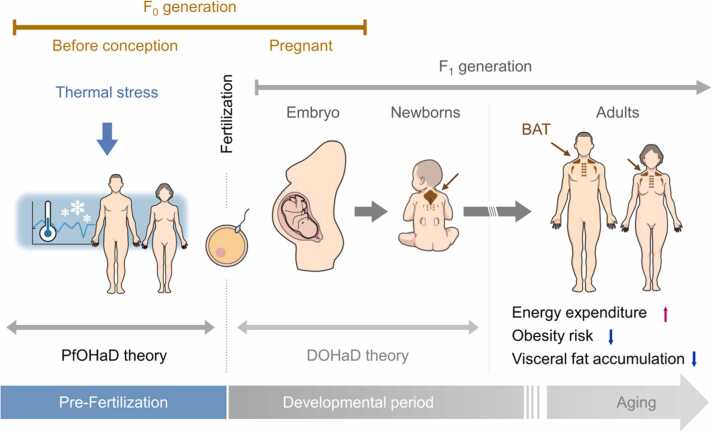
The transgenerational control of BAT activity and energy homeostasis by preconception cold exposure. Parental exposure to cold temperature and temperature gap preserves BAT activity to facilitate adaptive thermogenesis, thereby protecting against age-related obesity in humans. Our findings propose a conceptual theory, named PfOHaD, distinct from the Developmental Origins of Health and Disease (DOHaD) theory. Modified from Yoneshiro et al. [16].

Cellular plasticity and the energy demand of brown and beige adipocytes are regulated through transcriptional and epigenetic mechanisms [13], [14]. In this regard, the sympathetic nervous system plays a pivotal role in the regulation of the functions of both transcriptional factors and epigenetic enzymes [13]. Furthermore, clinical and preclinical evidence indicates that epigenetic predisposition induced by cold exposure prior to conception can be transmitted across generations, resulting in beneficial metabolic alterations [15], [16]. For instance, a pioneering study demonstrated in mice that paternal, but not maternal, cold exposure before conception enhances BAT thermogenesis and induces beige adipocyte formation in offspring possibly through mechanisms involving DNA methylation in sperm and sympathetic nervous system [15]. Utilizing the data of cold-induced BAT activity with the largest sample size, we found that human individuals whose mothers conceived during cold seasons exhibit higher BAT activity, increased EE, and lower body mass index, body fat content, and visceral fat area than individuals whose mothers conceived during warm seasons [16]. A spatiotemporal meteorological analysis revealed that the meteorological determinants of BAT activity and adiposity in the adulthood are lower outdoor temperatures and larger diurnal temperature gaps in a period before fertilization. These findings suggest that BAT metabolic fate and susceptibility of metabolic diseases are preprogrammed by the transgenerational epigenetic inheritance of preconception cold exposure in humans (Fig. 6). Although further validation in a larger population is necessary to substantiate these findings, an epidemiological study reported an increased cardiometabolic health in individuals who born in the autumn (the predicted season of fertilization is the winter) [17]. These findings serve to expand the traditional DOHaD theory by incorporating environmental conditions prior to fertilization, giving rise to the concept of Pre-fertilization Origins of Health and Disease (PfOHaD).

### What is the mediator of the transgenerational inheritance of cold memory?

Since transgenerational activation of BAT is paternal lineage-specific in mice [15], potential mediators include DNA methylation, retained histone modifications, and small RNAs in sperm [18], [19]. Notably, Sun *et al.* found that cold exposure induces hypomethylation of the β3 adrenergic receptor (*Adrb3*) gene coding region in the paternal sperm and increases *Adrb3* expression in the offspring BAT [16], suggesting the involvement of sympathetic nervous system in the transgenerational control of BAT. Interestingly, a recent study provides evidence of the discernible role of sperm-bone mitochondrial RNA, induced by paternal nutritional stress, in systemic glucose homeostasis of offspring [19]. It remains undetermined whether sperm-bone mitochondrial RNA mediates the transgenerational activation of BAT. Understanding of the molecular mechanisms by which preconception cold exposure activates BAT through environmental memory offers new therapeutic avenues for improving our health.

### Transgenerational control of BAT evokes an idea of exposome-mediated energy homeostasis

The apparent lifelong effects of cold exposure on BAT function provide another layer of idea; varied environmental exposure throughout a life, *a.k.a.* “Exposome”, may have long-term impact on BAT activity and its change with aging. Although the season of fertilization is one determinant of the metabolic fate of BAT, we observed a considerable individual variation of BAT activity, even among participants whose mothers conceived during cold seasons [16]. While most studies on BAT have used a single-exposure approach, such as cold exposure, the Exposome approach remains underutilized in BAT research. Researchers have recognized the need to consider the complex interactions between environments, lifetime, and metabolic homeostasis [20]. Understanding the role of Exposomes in controlling BAT, as well as the mechanisms responsible for preserving epigenetic memory, may be important for a clinical point of view for developing personalized predictive therapies that activate BAT and prevent metabolic disorders.

## Adaptive prioritization of environmental signals in real exposome conditions (Naoki Honda)

Living organisms in natural environments are exposed to multiple environmental signals that fluctuate simultaneously. Yet most experimental studies performed under tightly controlled laboratory settings cannot capture how organisms flexibly adjust their responses to these cues depending on context. To address this limitation, we analyzed long-term physiological and environmental records from medaka fish maintained outdoors, where temperature, day length, and solar radiation varied naturally over a two-year period [21].

Our data-driven approach estimates how organisms dynamically adjust the relative importance they assign to each environmental signal when regulating seasonal physiological processes such as gonadal development. We found that the importance of environmental cues varies over time, suggesting that organisms continually recalibrate which signals they treat as most informative for adaptation [22].

**Fig. 7:**
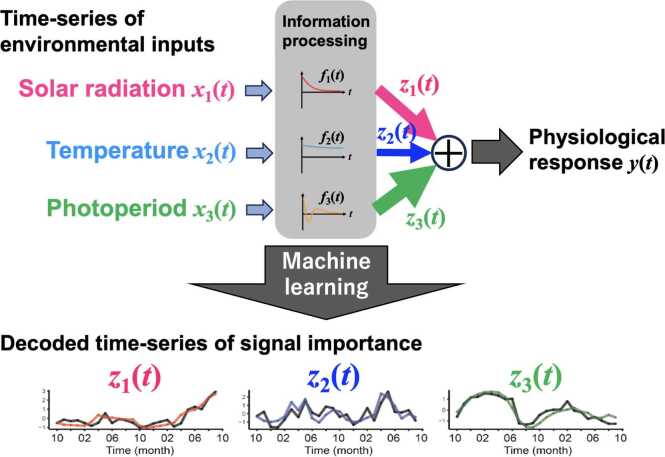
Adaptive prioritization of environmental signals under real Exposome conditions. Environmental inputs such as temperature, photoperiod, and solar radiation are dynamically weighted over time (w1(t), w2(t), w3(t)) to regulate physiological responses. The lower panel illustrates time-dependent changes in the relative importance of each environmental signal.

Comparisons with transcriptomic patterns further indicate that these shifts in environmental signal importance could be associated with temporal changes in gene expression. Thus, these findings highlight a complementary dimension of Exposome biology: not only what organisms are exposed to, but also how they differentially weight environmental information. Such insights underscore that the Exposome encompasses not only external exposures themselves but also the organism’s adaptive use of environmental information. Taken together, data-driven approaches provide new perspectives for understanding how such adaptive processes are regulated in natural environments (Fig. 7).

## Interdisciplinary research for infectious disease drug discovery through drug repurposing (Shingo Iwami)

The COVID-19 pandemic highlighted the threat of infectious diseases in an increasingly globalized world. It is crucial to use the lessons learned from this pandemic to prepare for future pandemics by establishing systems that can rapidly develop treatments for emerging infectious diseases. Although the development of new drugs typically requires substantial time and funding, the pandemic underscored the potential of "drug repurposing" as an approach to accelerate drug development in emergency situations. However, despite its promise, relatively few repositioned drugs were ultimately used as treatments, revealing several challenges that need to be addressed.

In this summary, I will provide an overview of our experiences with drug repurposing, clinical trial design, and the outcomes of these trials. We will discuss the key factors necessary to improve the success rate of drug discovery through repurposing and explore potential solutions. Additionally, I will touch on the collaborative research frameworks and interdisciplinary studies we are developing during peacetime to better prepare for the next pandemic.

Drug repurposing refers to the strategy of identifying new therapeutic indications for existing drugs. Unlike traditional drug development, which is time-consuming and costly, repositioning leverages prior knowledge of a compound’s safety, pharmacokinetics, and manufacturing processes. This approach can significantly reduce development costs and shorten the timeline from discovery to clinical use. Moreover, repositioned drugs often have well-documented safety profiles, which facilitates regulatory approval and clinical adoption. Drug repositioning has been particularly valuable during public health emergencies, such as COVID-19, when rapid identification of effective therapies is essential.

**Fig. 8:**
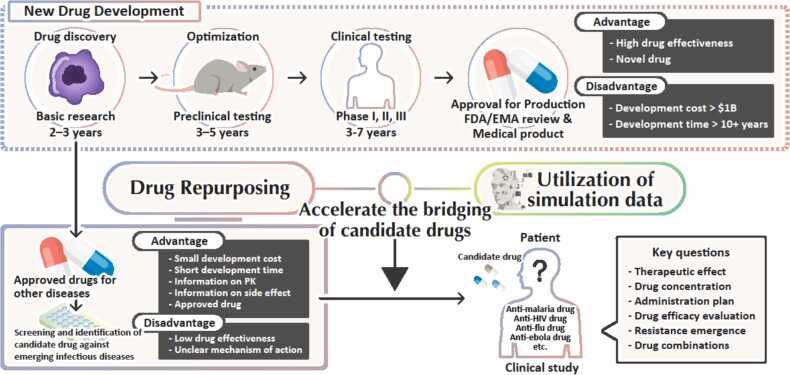
Drug development with drug repurposing approach and mathematical simulations.

In drug repurposing, comprehensive screening is often conducted as an initial step. Candidate compounds identified through screening are subsequently subjected to cell-based assays to characterize their antiviral activity in greater detail, typically yielding information such as drug response curves. However, predicting efficacy in patient solely from cell-based data remains challenging. Therefore, the integration of simulation approaches can facilitate the extrapolation of preclinical findings to the clinical setting and thereby accelerate the translation to clinical trials (Fig. 8).

During the COVID-19 pandemic, we conducted screening of more than 3000 compounds and identified several approved drugs that had not yet been tested in clinical trials but exhibited remarkably strong antiviral effects in cultured cells. Among these, nelfinavir (NFV) demonstrated particularly potent activity. Findings from cell-based infection assays, together with docking simulations, suggested that NFV could inhibit viral replication.

To elucidate the temporal dynamics of viral load with substantial inter-patient variability, we analyzed clinical data from SARS-CoV-2 patients using a nonlinear mixed-effects modeling approach. Time-course viral load data were used to estimate parameters of a within-host viral kinetics model, which reflect inter-patient variability. Based on these parameters, we reconstructed viral load trajectories and stratified patients, revealing distinct differences in the duration of viral shedding across subgroups.

Furthermore, by utilizing the estimated parameter distributions, we generated virtual patients through random sampling to perform simulations that account for inter-patient variability. We then calculated the sample size required to detect a statistically significant difference in the duration of viral shedding (time from symptom onset to viral load falling below the detection limit) when administering a hypothetical antiviral agent with 99% inhibitory efficacy. The analysis showed that enrolling all COVID-19 patients regardless of time since symptom onset would require more than 10,000 participants in both placebo and treatment arms. These findings underscore the importance of enrolling patients early after symptom onset to reduce sample size and improve trial feasibility.

In addition, to enable a more detailed prediction of the clinical efficacy of nelfinavir, we integrated the dose–response relationships obtained from cell-based experiments with pharmacokinetic data to calculate its inhibitory effect. Using the simulator explained in the previous part, we then calculated the required sample size for clinical trials. Based on these results, we determined the target sample size and successfully accomplished the translational step to clinical trials within four months.

However, the clinical trial showed no statistically significant difference in the primary endpoint, the duration of viral shedding. To explore potential reasons, we analyzed the discrepancy between drug efficacy observed in cell-based experiments and that achieved in patients. The analysis revealed that drug concentrations two- to five-fold higher than those effective in cell culture were required to achieve 50% inhibition of viral replication in patients. These findings indicate that future model-based predictions of therapeutic efficacy should account for the reduced activity observed in patients. Ideally, such estimates should be obtained before clinical trials, for example by analyzing data from compassionate use cases.

In conclusion, the strategy of combining drug repositioning with simulation is highly effective during pandemics caused by emerging infectious diseases. However, accurate prediction through simulation requires both a well-established infrastructure for data collection and the advancement of analytical methodologies that can fully exploit such data. Therefore, it is of critical importance to establish, during inter-pandemic periods, a sustainable preparedness framework for emerging infectious diseases, including strong collaboration between academia and industry.

## Future perspective

The studies presented in this symposium collectively highlight a shift from conventional environmental health models toward an integrated, systems-level understanding of biological responses to environmental complexity. Traditionally, environmental health has focused on linear relationships between single exposures and disease outcomes. In contrast, the approaches described here emphasize cumulative exposures (Exposome), biological encoding of environmental information (epigenetic and physiological memory), and predictive modeling enabled by Digital Transformation.

A central theme emerging from these studies is the continuity across biological scales. Environmental exposures are first encountered as Exposome, encoded at the molecular level through epigenetic modifications, manifested at the physiological level as long-term functional changes, and ultimately translated into disease risk or resilience. Digital approaches, including simulation and data-driven modeling, provide the necessary framework to integrate these layers and predict outcomes.

Importantly, these perspectives expand the scope of One Health beyond conventional environmental health paradigms. Rather than viewing humans, animals, and environments as separate domains linked by exposure, the integration of Exposome science and Digital Transformation enables a dynamic understanding of their interconnectedness. For example, transgenerational epigenetic inheritance links past environmental conditions to present health, while adaptive prioritization of environmental signals demonstrates how organisms actively interpret their environments.

Another key insight is the importance of temporal dynamics. Several studies highlighted the role of timing - particularly preconception and early-life exposures - in shaping long-term health outcomes. This temporal dimension remains underexplored in Exposome research and represents a critical area for future investigation.

Looking forward, the realization of One Health will depend on advances in three areas: (1) comprehensive measurement and prioritization of environmental exposures, (2) mechanistic understanding of how such exposures are encoded and retained, and (3) development of predictive models that can guide intervention strategies. Achieving these goals will require close collaboration across disciplines, including environmental science, biology, medicine, and data science.

Ultimately, the integration of Exposome science with Digital Transformation has the potential to transform One Health from a conceptual framework into a predictive and actionable science, enabling more effective prevention and management of disease across humans, animals, and ecosystems.

## Author contributions

Y.K., K.N., S.I., T.Y., N.H. were the speakers and M.N. and N.O. were the organizers in the symposium entitled “Interdisciplinary Cooperation of Digital and Exposome Researchers: Towards Comprehensive Understanding Interactions between Living Organisms and Environment (March 19th, 2025)” in APPW2025, the congress that was held in conjunction with 130th General Meeting of the Japanese Association of Anatomists, 102th General Meeting of the Physiological Society of Japan, and 98th Annual Meeting of the Japanese Pharmacological Society. All authors wrote approved of the manuscript.

## CRediT authorship contribution statement

**Nobuhiko Ohno:** Writing – review & editing, Writing – original draft. **Naoki Honda:** Writing – original draft. **Shingo Iwami:** Writing – original draft. **Keiko Nohara:** Writing – original draft. **Yoshito Kumagai:** Writing – original draft. **Takeshi Yoneshiro:** Writing – original draft. **Motohiro Nishida:** Writing – review & editing, Writing – original draft.

## Funding

This work was supported by Transdisciplinary & Exploratory Activity Momentum (TEAM) project from the Japanese Medical Science Federation. Y.K. receives funding from Grant-in-Aid for Scientific Research (S) (#25220103, 2013–2017) and (#18H05293, 2018–2022). T.Y. receives funding from the 10.13039/501100002241Japan Science and Technology Agency (JPMJFR2014), the JSPS KAKENHI (JP25K02698), and the 10.13039/100009619Japan Agency for Medical Research and Development (JP20gm1310007), and M.N. receives funding from the JSPS KAKENHI (26H02407).

## Declaration of Competing Interest

The authors declare that they have no known competing financial interests or personal relationships that could have appeared to influence the work reported in this paper.
